# The State of Art of Extracellular Traps in Protozoan Infections (Review)

**DOI:** 10.3389/fimmu.2021.770246

**Published:** 2021-12-14

**Authors:** Jing Zhang, Ying Sun, Jingtong Zheng

**Affiliations:** ^1^ Intensive Care Unit, First Hospital of Jilin University, Changchun, China; ^2^ Department of Pathogenobiology, College of Basic Medical Sciences, Jilin University, Changchun, China; ^3^ Department of Respiratory and Critical Care Medicine, First Hospital of Jilin University, Changchun, China

**Keywords:** extracellular traps, protozoan parasites, antimicrobial defense, NETs, METs

## Abstract

Protozoan parasite infection causes severe diseases in humans and animals, leading to tremendous economic and medical pressure. Natural immunity is the first line of defence against parasitic infection. Currently, the role of natural host immunity in combatting parasitic infection is unclear, so further research on natural host immunity against parasites will provide a theoretical basis for the prevention and treatment of related parasitic diseases. Extracellular traps (ETs) are an important natural mechanism of immunity involving resistance to pathogens. When immune cells such as neutrophils and macrophages are stimulated by external pathogens, they release a fibrous network structure, consisting mainly of DNA and protein, that can capture and kill a variety of extracellular pathogenic microorganisms. In this review, we discuss the relevant recently reported data on ET formation induced by protozoan parasite infection, including the molecular mechanisms involved, and discuss the role of ETs in the occurrence and development of parasitic diseases.

## 1 Introduction

Protozoa are unicellular organisms that can perform all the physiological functions required for life activities. There are approximately 10000 species of parasitic protozoa that live in the body or on the surface of animals, and some of these can be pathogenic (1). Among them, the common pathogenic protozoa include mainly *Plasmodium*, *Entamoeba*, *Leishmania*, and *Toxoplasma* species. Protozoan diseases seriously endanger the health of humans and other animals. Among them, malaria is the most widespread parasitic disease and the fifth most lethal parasitic infection in the world. According to the statistics of the World Health Organization (WHO), in 2018, there were 228 million malaria cases worldwide, approximately 405000 people died of malaria, and 67% of the deaths were children under the age of five (2). *Toxoplasma gondii*, as an obligate intracellular parasite, can infect most warm-blooded animals, including humans. The infection rate in some countries is as high as 40%. As toxoplasmosis is an opportunistic disease, approximately 80% of the primary infections are asymptomatic due to effective control by the host immune system. However, in infected people with low immune function, the pathogen can cross the placenta and cause adverse pregnancy outcomes and long-term birth defects (3). Unfortunately, resistance against some drug treatments is emerging (4). Therefore, an improved understanding of the role of immune cells in the resistance to protozoan disease is urgently required.

Immune cells such as monocytes, macrophages and neutrophils are considered to be the first line of defense against pathogen invasion (5). When immune cells encounter protozoa invading the body, they are activated to release extracellular traps (ETs). ETs are structures made up of intracellular components released by activated immune cells (such as neutrophils) that discharge DNA, histones and proteins derived from intracellular granules (6–9). In recent years, with the deepening of research, ETs have been indicated to kill a variety of parasites. For example, neutrophils can kill *Plasmodium* through ETs (10), and mast cell ETs can kill *Leishmania* (11). However, studies have found that ETs not only help cells resist pathogen invasion but also are related to the occurrence of a variety of diseases. ETs may aggravate the local inflammatory response by activating the complement and coagulation system, resulting in tissue damage (12).

Because knowledge of the special protection mechanisms of ETs is limited for protozoan parasite infection, this review will analyze recent findings regarding the correlations and differences among ETs whose formation is induced by different protozoan parasites (**Table 1**).

**Table 1:** Extracellular traps induced by different parasites and the associated signalling pathways.

Antigen	Cell source	Cell types that produce extracellular traps	Signalling pathway	Reduction in survival rate	Stimulation time	References
*Trypanosoma evansi*	Mouse	PMNs	ERK1/2	—	2 h	(13)
*Trypanosoma brucei*	Cattle	PMNs	–	26%	2 h	(14)
*Trypanosoma cruzi*	Human	PMNs	–	—	1 h	(15)
*Leishmania infantum*	Mouse and canine	PMNs	–	14.3-43%	2 h	(16, 17)
*Leishmania tropica*	Mouse and rat	Mast cells	Partially PI3K	13.56%	18-24 h	(11)
*Leishmania donovani*	Mouse and rat	Mast cells	–	9%	18-24 h	(11)
*Giardia duodenalis*	Mouse	Macrophages	ERK1/2 and p38 MAP	31.75%	2.5 h	(18)
*Trichomonas vaginalis*	Human	Macrophages	P38 and ERK1/2 MAPK	–	1 h	(19)
*Entamoeba histolytica*	Mouse	PMNs	–	17%	–	(20)
	Hamster	PMNs	–	–	–	(21)
*Naegleria fowleri*	Mouse	PMNs	–	–	–	(22)
	Human	PMNs	–	–	–	(23)
*Plasmodium falciparum*	Human	PMNs	–	–	3 h	(24)
*Besnoitia besnoiti*	Cattle	PMNs	P38 and ERK1/2 MAPK	34%	3 h	(25)
*Neospora caninum*	Canine	PMNs	ERK 1/2 and p38 MAPK	47%-52%	–	(26)
	Goat	Monocytes	ERK 1/2 and p38 MAPK	–	–	(27)
	Goat	PMNs	PI3K pathway	–	–	(28)
	Cattle	Macrophages	ERK 1/2 and p38 MAPK	–	–	(29)
	Cattle	PMNs	ERK 1/2 and p38 MAPK	–	–	(30)
*Toxoplasma gondii*	Mouse	PMNs	Raf-MEK-ERK	25%	4 h	(31)
	Human	PMNs	Raf-MEK-ERK	–	6 h	(31)
	Cat	PMNs	–	47%	3 h	(32)
	Dog	–	–	–	2 h	(33)

## 2 Phylum Sarcomastigophora, Class Zoomastigophorea

### 2.1 Trypanosoma

*Trypanosoma* is a genus of protozoa belonging to the phylum Sarcomastigophora, class Zoomastigophorea, order Kinetoplastida, and family Trypanosomatidae (34). *Trypanosoma evansi* (*T. evansi*) is a eukaryotic single-celled blood flagellate that is parasitic in almost all vertebrates (13). It can be transmitted between animals through vector flies and gadflies or directly through raw meat, infected animal blood or damaged mucosal tissues. *T. evansi* infects wild animals and livestock, causing animal *Trypanosoma* disease, also known as surra. The disease is widely distributed in tropical and subtropical areas (35). In recent years, *T. evansi* has also been reported to infect humans, suggesting that this pathogen has the potential to become a zoonotic parasitic pathogen (36). At present, there are few effective prevention and control methods for the disease. Therefore, as one of the important lines of defense against parasites, neutrophils may play an important role in the protection against this pathogen.

Recent studies have shown that *T. evansi* can be captured by ETs consisting of fine DNA fibres released from polymorphonuclear neutrophils (PMNs) (13). Mouse neutrophils stimulated with live or dead parasites can release reticular structures and attach themselves to the surface of the parasites, and the number of ETs formed is dependent on time and stimulant dose. Studies have shown that *T. evansi* can induce the production of free-radical reactive oxygen species (ROS) through ETs, and the production of ROS is also dose dependent. Because the activity of ROS kills the parasite and prevents invasion, it can be speculated that *T. evansi* can activate classic pathways that are ROS- and peptidylarginine deiminase 4 (PAD4)-dependent pathways and are related to the myeloperoxidase (MPO), neutrophil elastase (NE) and ERK1/2 signalling pathways (13).

In addition, *Trypanosoma brucei* (*T.brucei*), another African trypanosome, can also stimulate neutrophils and thus the release of ETs. Previous studies have shown that the parasite could induce the activation of bovine PMNs and the production of TLR2 and TLR4. TLR2 and TLR4 play a key role in the recognition and absorption of *T. brucei* spores, IL-8 production and neutrophil ET (NET) formation. The number of motile parasites was found to decrease by 26% due to the formation of NETs (14).

Another *Trypanosoma* species, *Trypanosoma cruzi*, can also cause ET formation. The infection time used in corresponding experiments was not 2 h but 1 h, which was inconsistent with the modelling time used for other trypanosomes (37). Different stimulation conditions could also lead to a reduction in the number of infected cells after treatment by the release of NETs induced by *T. cruzi*, but the number of parasites did not decrease.

In conclusion, NETs play a positive role in controlling parasitic infection. A recent study has shown that NETs can reduce the motility or infectivity of parasites without affecting the parasite viability of *T. evansi*, *T. brucei* and *T. cruzi in vitro*. Because motility is very important for the development and pathogenesis of parasites, the reduction in the number of motile parasites can still reflect the importance of NETs in controlling parasitic infection. In slightly different *in vivo* experiments in mice, treatment of *T. cruzi* with NETs significantly reduced the number of parasites in the blood (15).

### 2.2 Leishmania

*Leishmania* species are protozoa that belong to the phylum Sarcomastigophora, class Zoomastigophorea, order Kinetoplastida, and family Trypanosomatidae (4). *Leishmania* parasites can cause leishmaniasis, with the main hosts being vertebrates, including mainly rodents, *Canidae* species, and humans (38). To date, more than 88 countries in the world have reported cases of *Leishmania* infection, with 12 million people infected (39).

Since leishmaniasis is caused by the bite of sandflies on host skin, neutrophils in the blood pool are the most abundant leukocytes, and they are also the first batch of cells recruited to the infected site (40). Neutrophils play an immunomodulatory role by releasing NETs. NETs are released in response to *Leishmania* infection, but there are few reports on whether *Leishmania* can stimulate neutrophil activation and the release of NETs.

#### 2.2.1 Leishmania infantum

*Leishmania infantum* (*L. infantum*) is a pathogen species that causes visceral leishmaniasis and cutaneous leishmaniasis along the Mediterranean coast, *L. infantum*, has also been found in Xinjiang, China (41). Studies have shown that *L. infantum* promastigotes can effectively induce NET formation (16). The lipoxin A4 receptor is one of the receptors that mediates NET formation in neutrophils induced by *infantum* promastigotes. In addition, after blocking the lipoxin A4 receptor with the antagonist Boc, the release of NETs from neutrophils induced by *L. infantum* promastigotes decreased significantly. Moreover, the activator lipoxin A4 (LxA4) could partly induce the release of NETs (17).

In addition, different sources of neutrophils can also affect the release of NETs. Studies have shown that healthy neutrophils kill approximately five times as many parasites as neutrophils from infected dogs (14.3-43%). In addition, interestingly, in healthy dogs, the main function of neutrophils is phagocytosis. However, NETs are very important for the ability of neutrophils of naturally infected dogs to control *Leishmania* infection (16). Addition of DNase to the culture to disrupt NET structure significantly promoted the survival of parasites. In addition, to demonstrate the role of NETs in the elimination of *Leishmania*, recent studies have used DNase activity to destroy the structure of NETs. The results showed that the survival rate of the protozoa increased by 35% after DNase was added to neutrophils from dogs naturally infected with the protozoa (16).

#### 2.2.2 Leishmania tropica and Leishmania donovani

Since hypertrophy already exists in the skin and since mast cells are some of the first immune cells to encounter *Leishmania* promastigotes, mast cells are early reactive cells. Unlike neutrophils, mast cells phagocytize *Leishmania* and induce the release of inflammatory mediators. Previous studies showed that after mast cells were cocultured with *Leishmania tropica* (*L.tropica*) and *Leishmania donovani* (*L. donovani*) for 24 h, the mortality of these species increased to 13.56% and 9%, respectively (11). It has been noted that mast cells seem to be more sensitive to *L. tropica*. In addition, when *Leishmania* promastigotes were cocultured with mast cells, 11.7% of the mast cells phagocytized *Leishmania* promastigotes, and the phagocytosis was time dependent (11).

Since the phagocytosis of mast cells could be inhibited by cytochalasin D, the parasiticidal ability of ETs of mast cells could be measured by inhibiting the phagocytosis of mast cells in many kinds of parasites (11, 42–44). The results showed that the viability of *L. tropica* was significantly increased in mast cells cotreated with cytochalasin D compared to that with mast cell treatment alone. This result confirmed that the decrease in the viability of *L. tropica* may have been partly due to the phagocytosis of mast cells (42). However, after DNase was used to eliminate the effect of ETs, the viability of *L. tropica* was also significantly improved. Therefore, the findings suggested that ETs still have a certain killing effect on *Leishmania*.

Because *Leishmania* itself can regulate signalling pathway regulation in mast cells to escape immune activation, it may be difficult to study the signalling pathways involved in *Leishmania*-mediated induction of ET formation in mast cells. However, because *L. tropica* causes mast cell ET and ROS production (11), it is speculated that this species may induce the classic pathway and ROS-dependent pathway.

In conclusion, although *L. infantum*, *L. tropica* and *L. donovani* are all *Leishmania* species, the related mechanisms of ET induction caused by *Leishmania* spp. seem to be different. The ETs induced by *L. infantum* are mainly from neutrophils and can kill parasites. However, the ETs that are induced by and kill *L. tropica* and *L. donovani* are derived by stimulation of ET formation in mast cells. For *L. donovani*, the role of NETs is fixation of the parasite rather than killing of the parasite (45). This suggests that even parasites of the same genus may have different effects in the stimulation of ETs.

### 2.3 Giardia

#### 2.3.1 Giardia duodenalis

*Giardia* is a genus of protozoa belonging to the phylum Sarcomastigophora, order Diplomonadida, and family Hexamitidae. *Giardia* species mainly parasitize the human small intestine, gallbladder, and duodenum, causing giardiasis, and are among the common parasites causing human intestinal infection (46). These species are parasitic intestinal protozoans with a global distribution, infecting approximately 250 million people in developing countries (47). In addition to being endemic, the disease is also waterborne (48).

In the last 10 years, due to the development of tourism, the incidence rate among tourists has been high, so *Giardia* infection is also called tourism diarrhoea, which has attracted the attention of all countries. Moreover, in recent years, patients with acquired immunodeficiency syndrome (AIDS) have often been found to be infected with this disease (48).

*Giardia duodenalis* (*G. duodenalis*) is an extracellular parasitic protozoan. The formation of ETs by mouse macrophages induced by *G. duodenalis* and the killing effect of macrophage ETs (METs) on *G. duodenalis* remain unclear. The latest research results show that macrophages can defend against *Giardia* invasion by releasing METs, and the signalling pathways involved are the p38 and ERK pathways (18). This result is consistent with the ERK1/2 MAPK and p38 pathways being involved in macrophage-mediated ET formation induced by tachyzoites of *Neosporidium canis*. In addition, studies have shown that niacinamide adenine dinucleotide phosphate (NADPH) oxidase-guided oxidative metabolism is involved in the release of METs (18).

As has been proven for *Leishmania* and other protozoa, when coculturing with macrophages *in vitro*, special attention should be given to the role of phagocytosis because phagocytosis is the main method by which macrophages eliminate parasites. Therefore, to study the effect of METs on parasites, phagocytosis inhibition can be adopted to accurately determine the effect of ETs on the survival rate of parasites.

### 2.4 Trichomonas

#### 2.4.1 Trichomonas vaginalis

*Trichomonas vaginalis* (*T. vaginalis*) is a protozoan species belonging to the phylum Sarcomastigophora, order Trichomonadida, and genus *Trichomonas*. *Trichomonas vaginalis* is a parasite found in the human vagina and urinary tract (49). It causes a sexually transmitted infectious disease that may lead to *Trichomonas* vaginitis, urethritis, pelvic inflammation, premature birth and endometriosis (50).

The latest research has shown that in addition to protozoa such as *Neosporidia*, *T. gondii* and *Giardia*, *T. vaginalis* can also stimulate cells to produce ETs and release substances such as ROS to prevent pathogen invasion (19). Fei et al., 2019 showed that *T. vaginalis* could stimulate ET production by the human monocytic leukaemia cell line THP-1 (19). The ETs had typical structural characteristics. At the same time, the number of ETs was related to the ratio of *T. vaginalis* to THP-1 cells, and the number of ETs produced by *T. vaginalis*-stimulated THP-1 cells gradually decreased with time.

Moreover, the phosphorylation levels of the p38 MAPK and ERK1/2 MAPK signalling pathway components increased when THP-1 cells produced ETs, which proved that the ET formation stimulated by *T. vaginalis* was related to the p38 MAPK and ERK1/2 MAPK signalling pathways. This result is consistent with the ERK1/2 MAPK and p38 pathways being involved in ET formation induced by *Giardia* in macrophages. In addition, *T. vaginalis* stimulated THP-1 cells to produce IL-1β and TNF-α, which suggests that the ETs produced by *T. vaginalis*-stimulated THP-1 cells may be related to IL-1β and TNF-α (19). Many studies on the role of NETs have investigated the impact of NETs on the survival rate of parasites (14, 16), but there have been few studies on macrophages in this regard, so it remains challenging to clarify the role of macrophages in *T. vaginalis* infection.

Moreover, PMNs are the main immune cells that are considered to eliminate *T. vaginalis*. Therefore, some studies have tried to prove the relation between *T. vaginalis* and NETs. In 2018, studies showed that human neutrophils (without NETs) rapidly killed *T. vaginalis* in a dose- and contact-dependent manner (51). However, a recent study showed that *T. vaginalis* can induce NET formation (52). This is different from the results of a previous study. By comparing the two experiments, we found that differences in the experimental conditions of the two experiments may have led to the difference in the results. The first factor was the source of neutrophils. In the former experiment, human PMNs were used, while in the latter experiment, mouse PMNs were used. The second factor was the ratio of *T. vaginalis* to PMNs. In the former experiment, the ratio of *T. vaginalis* to human PMNs was 1:1, while in the latter experiment, the ratio of *T. vaginalis* to mouse PMNs was 2:1. The third factor was incubation time. In the former experiment, *T. vaginalis* was coincubated with human PMNs for 65 minutes, while in the latter experiment, *T. vaginalis* was coincubated with mouse PMNs for 2 h (51, 52). If the experimental differences were caused by the different sources of PMNs, that may indicate, remarkably, that neutrophils from different sources can produce different responses to the same parasite. In addition, in the latter experiment, although *T. vaginalis* induced NET formation by mouse PMNs, the findings did not specifically indicate the impact of PMNs on the viability of *T. vaginalis*, which is also a problem that needs attention.

## 3 Phylum Sarcomastigophora, Class Lobosea

### 3.1 Entamoeba

#### 3.1.1 Entamoeba histolytica

*Entamoeba histolytica* (*E. histolytica*) is a protozoan species belonging to the class Lobosea, order Amoebina, and family Amoebae. Amoebiasis caused by *E. histolytica*, which affects the intestine and liver, is a primary human intestinal infection (53). It is estimated that 50 million people are infected with *E. histolytica*. It causes 40-100 thousand deaths every year and is the third-leading cause of human death due to parasitic infection. There are two stages in the life cycle of *E. histolytica*: the cyst stage is the infection stage, and the trophozoite stage is the proliferation stage (54–56).

Díaz-Godínez (21) suggested that NADPH oxidase, which induces oxidative metabolism, was involved in the release of NETs. Later studies showed that calcium ionophores could induce the production of NETs; that is, the activity of PAD4 was also involved in the release of NETs (20, 57). However, these factors are not the key to the production of NETs stimulated by amoebae. Studies have shown that many parasites can inhibit the respiratory burst of neutrophils and easily induce NETosis (13, 28). For *E. histolytica*, the main factors causing NET formation are the MPO activity of histolytic trophozoites and neutrophils on the surface of parasites. After coming in contact with *E. histolytica*, neutrophils quickly transfer MPO to the surface of the *E. histolytica* and trigger ROS production, which is necessary to induce NET production (21).

Recently, it was found that the formation of reticular structures stimulated by *E. histolytica* is related to the neutrophil contact mode of *Entamoeba*. After contact with *E. histolytica*, neutrophils form NETs around the *E. histolytica* in an explosive manner and completely cover them in a fuzzy DNA structure. After that, the parasites are fixed and killed (58).

Although both mice and hamsters could be used for these experiments, differences in their NET and MPO levels were observed (20). *In vitro* experiments showed that mice produced more NETs and MPO than hamsters, whereas the elastase activity in both groups was very high. However, inhibition of NET formation and MPO activity promoted amoebal activity in mice. Mice showed higher levels of NETs and MPO than hamsters (20).

There exists a parasite that is similar to *E. histolytica* in both morphology and life history but not pathogenesis, i.e., *Entamoeba dispar* (*E. dispar*). Recent studies have shown that *E. dispar* does not induce the production of NETs (59). However, the underlying mechanism has not been discussed.

### 3.2 Naegleria

#### 3.2.1 Naegleria fowleri

*Naegleria fowleri* (*N. fowleri*) is a free-living amoeba that can infect humans through the nasal mucosa and cause central nervous system diseases, such as primary amoebic meningoencephalitis (PAM) (60, 61). Because NETs may be an important factor in the fixing and killing of invading microorganisms, the study of the role of NETs in central nervous system diseases caused by *N. fowleri* will help us to better understand the relevant mechanisms and regulatory pathways of parasites involved in the occurrence and development of diseases.

A recent study evaluated the ability of *N. fowleri* to induce the release of NETs from mouse PMN cells *in vitro* and *in vivo*. The results showed that when PMNs and *N. fowleri* were cocultured, the parasites could induce the release of NETs and MPO from the neutrophils in a time- and dose-dependent manner (22, 23). Because *N. fowleri* can infect humans through the nasal mucosa, if neutrophils are stimulated to release their ETs, *N. fowleri* may be damaged, and attachment to nasal mucosal cells may be prevented (22). In addition, studies have shown that NETs cannot damage non-IgG-opsonized *N. fowleri* trophozoites but can damage human IgG-opsonized *N. fowleri* trophozoites. Therefore, the findings suggested that IgG antibodies may play a role through NETs (23).

## 4 Phylum Apicomplexa, Class Sporozoasida

### 4.1 Plasmodium

*Plasmodium* is the causative pathogen of malaria and are harmful to human health (62). Approximately 50% of the global population is at risk of *Plasmodium* infection. Despite great efforts to eradicate malaria worldwide, approximately 228 million malaria cases were reported in 2018 (63). The genus *Plasmodium* belongs to the phylum Apicomplexa, class Sporozoasida, and order Haemosporida. The common malaria parasites infecting humans include mainly *Plasmodium vivax*, *Plasmodium malariae*, *Plasmodium falciparum* and *Plasmodium ovale* (64).

#### 4.1.1 Plasmodium falciparum

In 2008, Virginia S Baker and others first found that malaria can cause NET formation, and many studies have focused on NETs (24). Recent studies have shown that *Plasmodium falciparum* (*P. Plasmodium*) infection can cause erythrocyte rupture (65). When erythrocytes lyse, they release haem crystals into the blood circulation. These haem crystals are usually taken up and removed by neutrophils. The main scavenging methods are closely related to NETs (65). Other studies have shown that neutrophils scavenge haemozoin crystals in the extracellular space through endocytosis and phagocytosis of vesicles rather than through the release of NETs. Human plasma components such as fibrinogen limit the clearance of haemozoin crystals, while the presence of platelets enhances the clearance of haemozoin crystals (66). These results show that the role of neutrophils may be multifaceted and may be related to the neutrophil source.

To test the effect of NETs on malaria, Rodrigues DAS cultured *P. falciparum* -infected erythrocytes in NET-rich supernatant (67). The results showed that the presence of NETs resulted in a significant decrease in the proportion of ring structures and infected erythrocytes in culture. In addition, studies have shown that parasites in erythrocytes produce uric acid. After the rupture of infected cells, the uric acid is released simultaneously with monosodium urate (MSU) crystals and crystalline haem. MSU crystals can induce the formation of intravascular NETs. Haem pigment activates neutrophils and fixes parasites and crystals on the endothelium (68). Furthermore, NETs protect endothelial cells from the proinflammatory effects of MSU (69). Moreover, after children are infected with *P. falciparum*, NETs appear with attached parasites and erythrocytes, and the level of antinuclear IgG antibodies (ANA) increases; Th2 cytokines are dominant in this process (65), which suggests the existence of a protective effect of NETs. Erythrocytes, *P. falciparum* and isolated human neutrophils were cocultured to show that *P. falciparum*-infected erythrocytes release macrophage migration inhibitory factor (MIF), which leads to the formation of NETs in neutrophils. The mechanism depends on C-X-C chemokine receptor type 4 (CXCR4). The production of NETs was dependent on time and parasite dose and was related to c-Jun N-terminal kinase (JNK) and PAD4 but not to ROS, neutrophil elastase(NE), MPO or p38 (67).

Although NETs play a positive role in antiparasitic infection, the role of NETs may be two-sided. Studies have shown that haem-induced NETs are necessary for the pathogenesis of malaria. The use of patient samples and mouse models showed that although soluble ET components could promote parasite isolation (68), they could also mediate tissue destruction, leading to the onset of malaria (70). Studies have shown that NETs can inhibit the reproduction of parasites infected by asymptomatic *P. falciparum*, but neutrophil activation and NET release may be one of the pathogeneses of severe *P. falciparum* infection (71). Therefore, neutrophils play a key role in malaria immunopathology.

In addition, to clarify the function of NETs in *P. falciparum* infection *in vivo*, Knackstedt et al. used Ne/PR3-/- mice as a NET defect model (72). In addition, DNase 1 -/- mice were also used in the experiment, in which NETs were generated as normal in the absence of DNase 1 but persisted at the release site because they were not processed by soluble DNase 1 in these mice. The results showed that after *Plasmodium* infection, the WT group exhibited liver damage and immunopathology, but the livers of the infected Ne/PR3-/- and DNase 1 -/- groups was not affected, and these groups showed no difference from the uninfected group. When NETs were injected into Ne/PR3-/- mice *in vitro*, liver injury was observed in parasitized mice, similar to the WT group (72). This result proves the direct pathogenicity of NETs.

In conclusion, in the face of *P. falciparum* infection, we can preliminarily hypothesize that the formation and activation of NETs is a double-edged sword. On the one hand, the NETs form a barrier on the surface of endothelial cells to protect endothelial cells from haem crystal-induced damage (69). On the other hand, they also mediate tissue destruction and limit the perfusion of terminal organs. Inflammatory mediators released during the coupling of NET formation and coagulation lead to the opening of the neuroimmune blood-brain barrier. In severe cases, this effect leads to cerebral malaria (73).

### 4.2 Besnoitia

#### 4.2.1 Besnoitia besnoiti

*Besnoitia besnoiti* (*B. besnoiti*) belongs to the phylum Apicomplexa, class Sporozoasida, and order Eucoccidiorida and usually parasitizes cattle, cats, horses, antelopes, deer and camels (74). After cattle swallow sporified oocysts of this species, sporozoites are released and enter the blood circulation through the gastrointestinal mucosa. They germinate in vascular endothelial cells, especially in the dermis, subcutaneous tissue, fascia and upper respiratory tract mucosa, producing a large number of tachyzoites. Then, the tachyzoites escape from ruptured cells, repeatedly invade other cells, continue to proliferate, and finally are enter connective tissue to form cysts due to environmental factors (74). At this time, the tachyzoites in the capsule become bradyzoites. Because the disease causes weight loss, a decline in milk production, temporary or permanent infertility of cows, and a long course of disease, it has caused tremendous economic losses in local cattle industries (25).

Because the pathogenicity of parasites is related to the continuous infection and proliferation cycle of target cells, NET-mediated parasite capture and inhibition of host cell invasion play a very important role in the occurrence and development of diseases. It has been reported that *B. besnoiti*-mediated NET formation seems to be NADPH oxidase (NOX)-and NE-MPO dependent and can effectively prevent tachyzoites from invading active host cells (75).

After bovine neutrophils and tachyzoites were coincubated for different durations and at different doses, parasite-induced NET formation was found to be time and dose dependent (76). When neutrophils were exposed to live parasites, UV-weakened parasites and tachyzoite homogenate, all of them showed obvious induction of NET formation. After DNase enzyme treatment, neutrophils were incubated with NOX, NE and MPO inhibitors, and NETs were eliminated (76). The findings indicate that NET formation occurs through the RAF-MEK-ERK signalling pathway and involves the activation of p38 MAPK.

Anja Taubert’s research team showed that when tachyzoites were mixed with neutrophils at 3:1 for 3 h, approximately 1/3 of the tachyzoites were fixed in NETs, and NET formation hindered the invasion by tachyzoites of host cells, leading to a 40% reduction in the infection rate (25). Notably, bovine NETs induce injury in infected host endothelial cells. Host cell injury leads to significant changes in the diameter and number of parasitic vacuoles during the development of intracellular parasites, but it does not affect the proliferation of parasites over time (75).

While *B. besnoiti*-mediated NETs are well recognized, *B. besnoiti* can also induce ET formation in monocytes. It has been reported that the number of ETs is dependent on time and stimulus dose. Carlos Hermosilla’s research team showed that monocyte-derived ETs were effectively eliminated by DNase I treatment and significantly reduced by treatment with MPO and NOX inhibitors, suggesting the key role of ROS and MPO in monocyte formation (77).

### 4.3 Neospora

#### 4.3.1 Neospora caninum

*Neospora caninum* (*N. caninum*) belongs to the class Aconoidasida and order Eucoccidia. It is an obligate intracellular protozoan parasite that causes serious reproductive disorders in ruminants worldwide (78). *N. caninum* can infect a wide range of hosts. In the life cycle of *N. caninum*, there are two distinct development modes: sexual reproduction and asexual reproduction. Sexual reproduction occurs only in the final canid hosts. These animals can transmit *N. caninum* (79). Therefore, sexual reproduction is related to the epidemiology of the disease. On the other hand, asexual reproduction seems to occur in many intermediate hosts, such as cattle, sheep, and rabbits. Therefore, ETs associated with *N. caninum* can be derived from a variety of animal neutrophils and other monocytes (28, 30, 80).

PMNs fight against a variety of invasive pathogens by releasing NETs and *via* other different mechanisms (6, 7, 9, 12). Studies have shown the interaction of goat neutrophils and *N. caninum in vitro*. Scanning electron microscopy and immunofluorescence analysis of tachyzoites of *N. caninum* showed that goat neutrophils exposed to *N. caninum* after NETosis released prominent filaments to trap parasites (28). In addition, the prominent filaments induced by *N. caninum* tachyzoites showed typical DNA and proteins, confirming the molecular characteristics of classic mammalian NETs (28).

Interestingly, some studies have proven that the NETs of goat neutrophils induced by *N. caninum* seem to employ a regulatory mechanism different from the signalling pathway involved in NET formation by canine neutrophils (26, 28). The signalling pathways associated with NETs of canine neutrophils are the ERK1/2 and p38 MAPK pathways. Although NETs from goat neutrophils induced by *N. caninum* were affected by MPO, they were not related to the activities of NOX, store-operated calcium entry (SOCE), ERK1/2 or p38 MAPK. The production of NETs was independent of stimulation time and dose (26). In addition, similar to NETs whose formation was induced by bacterial infection, pentraxin also appeared to play a role in the NET formation process of goat neutrophils induced by *N. caninum*.

In fact, *N. caninum* can cause not only NET formation but also the release of ETs by monocytes (27). Macrophages are multifunctional phagocytes that are considered irreplaceable in the early natural immune response of the host to microbial and parasitic pathogens (29). Yang (27) first studied the effect of *N. caninum* tachyzoites on the release of ETs from goat monocytes and further clarified some of the underlying molecular mechanisms. The formation of monocyte-derived ETs induced by tachyzoites was observed by scanning electron microscopy. Changes in H3 histone and MPO in the structure of monocyte ETs were observed by laser scanning confocal microscopy. The results showed that tachyzoites could trigger the formation of ETs in goat monocytes and that the ETs released by monocytes could embed live tachyzoites. Histone and MPO modified the DNA in the structure of monocyte-derived ETs, which indicated the presence of the classic components of ETs. In addition, inhibitors of NOX oxidase, MPO, ERK1/2 or the p38 MAPK signalling pathway significantly reduced tachyzoite-induced goat monocyte-derived ET formation (27). The results were similar to the release of ETs from goat monocytes induced by *N. caninum* tachyzoites. This is the first report on the secretion of ETs by goat monocytes after exposure, suggesting that this early innate immune effect mechanism may be related to the acute stage of goat neosporidiosis.

### 4.4 Toxoplasma

#### 4.4.1 Toxoplasma gondii

*Toxoplasma gondii* (*T. gondii)*, belonging to the class Aconoidasida and order Eucoccidia (81), is an intracellular parasite that enters blood circulation to reach all parts of the body and destroys the brain, heart and fundus, resulting in a decline in human immunity and affliction with various diseases. The life cycle of *T. gondii* requires two hosts: the intermediate hosts include reptiles, fish, insects, birds, mammals and humans, and the final host is Felidae species (82). In recent years, with economic growth and the continuous improvement in people’s living standards, pets have become increasingly popular. However, due to the complexity of the pet market, pets have become important disease carriers (83). Moreover, this situation has led to an increase in the *T. gondii* infection rate among Chinese residents. It is estimated that approximately one-third of people worldwide are infected with *T. gondii*. The average *T. gondii* infection rate of humans in China is 7.88%, and that of animals may be higher (84).

Although *T. gondii* and *B. besnoiti* belong to the same family, Sarcosporididae, the roles of ET formation mediated by *T. gondii* and *B. besnoiti* in inhibiting parasite invasion in host cells seem to be different (31, 32, 75, 76). *B. besnoiti* plays an important role in ET-mediated parasite capture and inhibition of parasite invasion (75, 76). However, studies have shown that although neutrophils produce ETs that can kill *T. gondii*, *T. gondii* can stimulate neutrophils in mice to produce ETs without invading host cells (31). That is, there is no absolute correlation between the ability to produce ETs and the invasion ability of parasites. Studies have shown that ETs produced by mouse neutrophils can kill 25% of *T. gondii* tachyzoites, which shows that ETs can effectively control *T. gondii* infection and play an important role in innate immunity (31). However, in studies in which neutrophils were stimulated with *T. gondii* to induce the production of ETs, although it was shown that dog neutrophils producing ETs could capture and kill *T. gondii*, the specific number and proportion of killed *T. gondii* tachyzoites were not clearly described (33). Quantitative experiments showed that the release of ETs triggered by *T gondii* tachyzoites was time independent and that the increase in NET release decreased significantly after 120 minutes (33). *T. gondii* tachyzoites could also escape ETs after 90 minutes, but the relevant mechanism needs to be further elaborated.

In the study of the relevant signalling pathways, the formation of ETs by *T. gondii*-stimulated mouse and human neutrophils was also found to be related to the Raf-MEK-ERK signalling pathway, because inhibiting this pathway reduced the production of ETs (31). The ROS or MAPK signalling pathway is related to the process by which *T. gondii* stimulates dog neutrophils to produce ETs (33). However, the signalling pathways involved in cat neutrophils seem to be different, and selective inhibitors blocking PI3Kδ could reduce the infection of cat neutrophils with *T. gondii*, while selective inhibitors blocking PI3Kγ had no impact on NETs (32). The central granulocytes in cats are not regulated by the ERK1/2 signalling pathway, which may be related to the species from which the neutrophils originated.

## 5 Overview of Extracellular Traps

Since it was first described that parasites can stimulate ET formation in 2008, research on ETs has progressed rapidly. Studies have shown that a variety of parasites can induce ET formation, but at present, there seems to be no relatively standardized process for the modelling and detection methods. Therefore, exploring the experimental methods for ET induction is important.

The first factor is the incubation time. To prove that the ability of a parasite to stimulate ET formation is time dependent and dose dependent, the coincubation time was examined, and the coincubation time of most neutrophils or macrophages with parasites was more than 2 h. However, over time, the formation of ETs may gradually reach a bottleneck rather than increase linearly.

The second factor is the incubation ratio. To ensure that ETs could be clearly detected, the gradient dilution method was used. The incubation ratio between most cells and parasites ranged from 1:1 to 1:5, but most experiments used for SEM detection and ET quantification use a ratio of 1:3 or 1:4. The reason for this discrepancy may be that if the incubation ratio between cells and parasites is too low, the effects of ETs may not be obvious.

In addition, Transwell assays have been applied in ET experiments. To confirm whether the death of pathogens is related to direct contact with immune cells such as macrophages, coculture of pathogens and immune cells in a Transwell system is a good method. In particular, since it is known that neutrophils can phagocytize and release NETs, NETs are often identified by the final reduction in live parasite levels, subconsciously ignore the possible role of phagocytosis. Cytochalasin D can be used to inhibit phagocytosis, but Transwell assays can also be used to directly prevent phagocytosis. In the previous discussion, it was mentioned that some neutrophils destroyed some pathogens mainly through phagocytosis, while some other pathogens were captured and destroyed by NETs. Transwell experiments confirmed that this phenomenon may be related to the size of the pathogen itself. In other words, even if the pathogen itself can induce the production of NETs, due to volume, neutrophils process these pathogens by phagocytosis rather than NET production. In addition, parasites such as *Trypanosoma* (85), *Entamoeba* (86), *Giardia* (87), *Leishmania* (88), *T. vaginalis* (89) and *Eimeria* (90) that may carry viruses themselves may affect the pathway of ET formation in neutrophils and may affect the results. Therefore, the application of Transwell assays could help us better study the real role of immune cells in the future.

## 6 Conclusion

In summary, in this paper, we discussed the ability of several different parasites, such as *Trypanosoma*, *Entamoeba*, *Plasmodium*, *Giardia*, and *Leishmania*, to induce ET formation and the molecular mechanisms involved. For example, *Trypanosoma* can stimulate neutrophils and cause the release of NETs, which involved ROS- and PAD4-dependent pathways and was related to the MPO, NE and ERK1/2 signalling pathways. *L. tropica* can stimulate mast cells to produce ETs associated with ROS-dependent pathways. *G. duodenalis* can stimulate macrophages that defend against *Giardia* by releasing METs, and the signalling pathway involved are the p38 and ERK pathways. Most of the time, ETs play a positive role in combating parasitic infection. However, the effect of ETs on *Plasmodium* spp. is complex, for example it may be one of the pathogeneses of severe *P. falciparum* infection. Therefore, clarifying the effect of ETs on parasites may play an important role in the development of antiparasitic drugs.

## Author Contributions

JTZ performed most of the research and data analyses and helped draft the manuscript. JZ and YS analyzed and interpreted the raw data. All authors read and approved the final manuscript.

## Funding

This work was supported by the Bethune Research Plan of Jilin University (grant number: 2020-31), and the training plan for Lixin excellent young teachers of Jilin University (grant number: 2021) to JTZ.

## Conflict of Interest

The authors declare that the research was conducted in the absence of any commercial or financial relationships that could be construed as a potential conflict of interest.

## Publisher’s Note

All claims expressed in this article are solely those of the authors and do not necessarily represent those of their affiliated organizations, or those of the publisher, the editors and the reviewers. Any product that may be evaluated in this article, or claim that may be made by its manufacturer, is not guaranteed or endorsed by the publisher.
